# The Use and Effects of an App-Based Physical Activity Intervention “Active2Gether” in Young Adults: Quasi-Experimental Trial

**DOI:** 10.2196/12538

**Published:** 2020-01-21

**Authors:** Anouk Middelweerd, Julia Mollee, Michel MCA Klein, Adnan Manzoor, Johannes Brug, Saskia J te Velde

**Affiliations:** 1 Department of Epidemiology and Biostatistics Amsterdam Universitair Medische Centra Vrije Universiteit Amsterdam Amsterdam Netherlands; 2 Vrije Universiteit Amsterdam Amsterdam Netherlands; 3 National Institute for Public Health and the Environment Ministry of Health, Welfare and Sport Bilthoven Netherlands; 4 Research Center for Healthy and Sustainable Living Hogeschool Utrecht University of Applied Sciences Utrecht Utrecht Netherlands

**Keywords:** physical activity, smartphone, mobile app

## Abstract

**Background:**

Insufficient physical activity (PA) is highly prevalent and associated with adverse health conditions and the risk of noncommunicable diseases. To increase levels of PA, effective interventions to promote PA are needed. Present-day technologies such as smartphones, smartphone apps, and activity trackers offer several possibilities in health promotion.

**Objective:**

This study aimed to explore the use and short-term effects of an app-based intervention (Active2Gether) to increase the levels of PA in young adults.

**Methods:**

Young adults aged 18-30 years were recruited (N=104) using diverse recruitment strategies. The participants were allocated to the Active2Gether-Full condition (tailored coaching messages, self-monitoring, and social comparison), Active2Gether-Light condition (self-monitoring and social comparison), and the Fitbit-only control condition (self-monitoring). All participants received a Fitbit One activity tracker, which could be synchronized with the intervention apps, to monitor PA behavior. A 12-week quasi-experimental trial was conducted to explore the intervention effects on weekly moderate-to-vigorous PA (MVPA) and relevant behavioral determinants (ie, self-efficacy, outcome expectations, social norm, intentions, satisfaction, perceived barriers, and long-term goals). The ActiGraph wGT3XBT and GT3X+ were used to assess baseline and postintervention follow-up PA.

**Results:**

Compared with the Fitbit condition, the Active2Gether-Light condition showed larger effect sizes for minutes of MVPA per day (regression coefficient B=3.1; 95% CI −6.7 to 12.9), and comparatively smaller effect sizes were seen for the Active2Gether-Full condition (B=1.2; 95% CI −8.7 to 11.1). Linear and logistic regression analyses for the intervention effects on the behavioral determinants at postintervention follow-up showed no significant intervention effects of the Active2Gether-Full and Active2Gether-Light conditions. The overall engagement with the Fitbit activity tracker was high (median 88% (74/84) of the days), but lower in the Fitbit condition. Participants in the Active2Gether conditions reported more technical problems than those in the Fitbit condition.

**Conclusions:**

This study showed no statistically significant differences in MVPA or determinants of MVPA after exposure to the Active2Gether-Full condition compared with the Active2Gether-Light or Fitbit condition. This might partly be explained by the small sample size and the low rates of satisfaction in the participants in the two Active2Gether conditions that might be because of the high rates of technical problems.

## Introduction

Insufficient physical activity (PA) is associated with adverse health conditions and noncommunicable diseases such as cardiovascular diseases, cancer, and diabetes [1,2]. Worldwide, approximately 25% of the adult population does not meet the recommended guidelines for PA [3]. In Western countries such as the United States and the Netherlands, approximately 50% of the population does not meet the guidelines [4]. Moreover, engagement in moderate-to-vigorous PA (MVPA) decreases with age, in particular, when transitioning from adolescence to (young) adulthood [5,6].

To increase the levels of PA, effective interventions to promote PA are needed. Research has shown that interventions are more likely to be effective when established behavior change techniques, such as self-monitoring, goal setting, and providing feedback on performance, are incorporated [7]. Systematic reviews further showed that individually tailored interventions are superior to generic interventions in promoting PA in effects as well as user engagement and appreciation [8-14]. Moreover, Krebs et al [10] demonstrated that dynamic tailoring (ie, iteratively assessing and providing feedback) was associated with larger effect sizes compared with static tailoring (ie, all feedback is based on one baseline assessment).

Present-day technologies such as smartphones, smartphone apps, and activity trackers offer possibilities to deliver theory-based, dynamically tailored interventions that include effective behavior change techniques. The high adoption rate of smartphones (97% among adults aged 20-29 years in the Netherlands) and the popularity of health and fitness apps and activity trackers [15] suggest that young adults may appreciate and adopt app-based PA interventions. Moreover, systematic reviews show that app-based interventions show promising results on changing health behavior, including PA [16-18]. Furthermore, the majority of interventions that reported significant changes in behaviors and health-related outcomes included behavior change techniques such as goal setting, self-monitoring, and feedback on the performance [16].

In this context, we developed the *Active2Gether* intervention. A systematic and stepwise approach was used to develop the Active2Gether intervention guided by health behavior theory and scientific evidence [19]. This resulted in the development of an app suitable for providing highly tailored coaching messages that are framed in an autonomy-supportive style. These coaching messages include behavior change techniques aiming to address relevant behavioral determinants (ie, self-efficacy, outcome expectations, intentions, impediments, long-term goals, social norm, satisfaction, and self-regulation skills) and are partly context specific. A fundamental component of the intervention is the model-based reasoning engine, that is, a software system that generates conclusions from information stored in the database using logical techniques and a mathematical model that is used to predict behaviors by computer simulations. The reasoning engine is used to tailor the intervention with respect to the type of support provided by the app, to send relevant and context-specific messages to the user, and to tailor the graphs displayed in the app. Detailed information on the development and technical design of the Active2Gether intervention can be found elsewhere [19,20].

The primary objective of the Active2Gether intervention was to increase the total time spent in MVPA for participants who do not meet the Dutch guidelines, to maintain PA levels of those who meet the guideline, or to further increase the PA levels if they indicate that they want to improve further. The secondary aims were (1) to increase the underlying specific categories of MVPA, that is, minutes of weekly sports participation, weekly numbers of stairs climbed, or weekly minutes of active transport and (2) to enhance the underlying determinants of the PA behaviors.

The aim of this study was to explore the use and effects of the Active2Gether intervention on increased weekly levels of MVPA and psychosocial determinants of MVPA in adults aged 18-30 years compared with two control groups in a quasi-experiment. As we could not achieve a sufficiently valid and powered research design, this paper is an exploratory study.

## Methods

### Design

A three-arm quasi-experimental trial was conducted to evaluate the short-term effects of the Active2Gether intervention. The trial included baseline, mid-intervention (6 weeks), and postintervention assessments. Data were collected between March 2016 and October 2016. The trial was registered (Dutch Trial Registry registration number NTR5630), and the project protocol was approved by the ethics committee of the VU Medical Center, Amsterdam. All participants provided written informed consent. The development of the Active2Gether intervention and evaluation plan are described in more detail in an earlier publication [19].

### Participants

Young adults were recruited by flyers, posters, social media, personal contacts, and snowball strategies. The majority of the participants were recruited through social media (48.4%, 42/104), other participants (23.1%, 24/104), and flyers and advertisement (11.5%, 12/104) in the regions of Amsterdam, Leiden, and Utrecht in the Netherlands.

**Figure 1:**
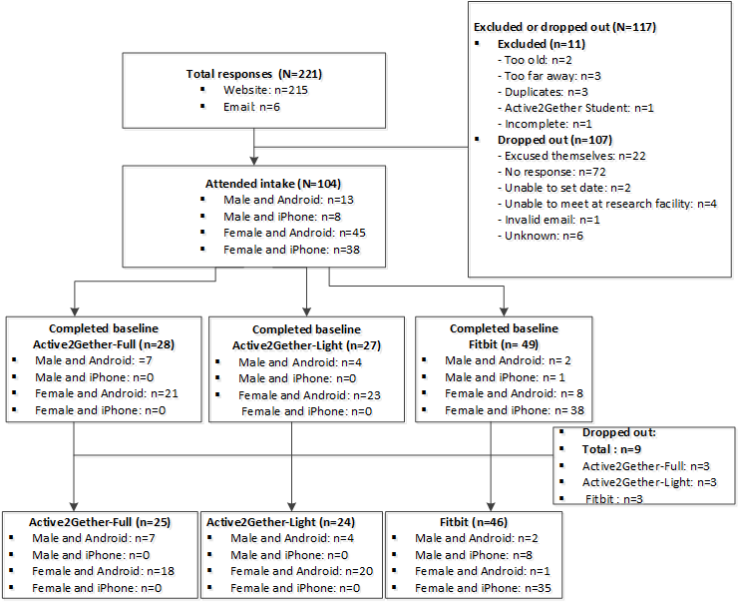
Flow diagram of the participants that were excluded or dropped out.

Participants registered for the trial through the Active2Gether website by completing a Web form asking information about gender, age, and type of smartphone they owned (ie, Android [Google Inc] or iOS [Apple Inc]). Regarding eligibility criteria, participants were considered eligible for the study if they were (1) aged 18-30 years at the time of registration, (2) in possession of a suitable smartphone running on Android or iOS, (3) apparently healthy, (4) Dutch speaking, and (5) signed the informed consent form. Participants were excluded if they were unable to visit the research facilities for the intake procedure. Figure 1 shows a flow diagram that outlines the reasons for exclusion or withdrawing from the study.

### Group Allocation

Stratified group allocation was applied, stratified by type of smartphone and gender. As the Active2Gether app only runs on Android, iPhone users were automatically assigned to the Fitbit condition, whereas Android phone users were randomly allocated to one of the 2 Active2Gether conditions after stratification by gender. The aim was to divide men and women with an Android phone equally over the 2 Active2Gether conditions and to allocate friends to the same condition. This was done by applying a 1:1 ratio to the order of registration. As a result, one Android user was allocated to the Fitbit condition. Randomization of Android users after gender stratification was performed before the participants visited the research facilities.

### Intervention

As described above, the participants were allocated to one of the three conditions: (1) the Active2Gether*-Full* condition, (2) the Active2Gether*-Light* condition, and (3) the *Fitbit* condition.

The participants in the Active2Gether*-Full* condition received an Android app that provided tailored advice aiming to increase weekly levels of MVPA. For this purpose, participants were coached on sports participation, taking the stairs, or active transport. Every week, the participants were asked to choose their coaching domain and set a weekly goal. Participants received a suggestion for a coaching domain and a weekly goal based on their previous behavior, but the final decision was up to the user. The participants received a Fitbit One (Fitbit Inc) activity tracker that could be synchronized to the app and allowed the participants to monitor their PA behavior. The app sent (daily) coaching messages addressing relevant behavioral determinants, that is, self-efficacy, outcome expectations, intentions, satisfaction, barriers, and self-regulation skills. The content of the messages was tailored to the user’s behavioral determinants, occupational status, and weather. The participants could receive up to three messages a day. Finally, the app displayed the activity data of the participant, including a graph displaying the activity data of six other participants, preferably friends. The graph with the activity data of others ranked the participants based on their weekly step activity and the user’s preferences for social comparison, that is, upward or downward comparison. Detailed information on the development and the technical design of the Active2Gether intervention can be found elsewhere [19,20].

The participants in the Active2Gether*-Light* condition received a slimmed-down version of the Active2Gether-Full app. Similar to the Active2Gether-Full condition, the participants received a Fitbit One tracker that could be synchronized to the app and allowed the participants to monitor their PA behavior. In addition, activity data of six other participants were shown in the same way as in the Active2Gether-Full condition. However, this variant of the Active2Gether app did not send tailored coaching messages.

The participants in the *Fitbit* condition only received a Fitbit One tracker and the Fitbit app. The Fitbit app is a publicly available—compatible with iPhones and Android phones—and enabled participants to monitor their step activity and set activity goals, that is, goals for the number of steps and flights of stairs [21]. Participants did not receive weekly emails (with a weekly summary of the progress and congratulations on earning badges) that Fitbit sends to its users.

### Procedure

A total of three rounds of assessments were conducted: at baseline, at 6-week follow-up (mid-trial), and after completion of the 12-week intervention period. For the majority of the participants, the postintervention measurement was delayed because of absence during the summer holidays. Participants completed a Web-based questionnaire at all points and wore an ActiGraph accelerometer at baseline and postintervention follow-up, providing objective measurements on the levels of PA.

After registering through the Active2Gether website, participants received an email providing detailed information about the study. Participants were asked to visit the research facilities once for an intake of about 1 hour. During the intake, participants again received detailed information about the study, and they signed an informed consent form, completed the baseline survey, installed the app(s) that were needed, and received a Fitbit One tracker. To complete the baseline measurements, participants were asked to wear an ActiGraph accelerometer for 1 week to objectively assess their baseline PA levels. During that week, no coaching messages were sent. After 6 weeks, participants received an automatically generated email inviting them to complete the online follow-up questionnaire. At the end of the study, after 12 weeks, participants were asked to complete the final online questionnaire (the link was automatically sent after 12 weeks) and to wear the ActiGraph accelerometer for another week. The participants did not have to visit the research facilities for the 6-week and postintervention follow-up assessments: After 6 weeks, the participants received an email with a link to the 6-week follow-up questionnaire; and after 12 weeks, participants received an email with a link to the postintervention follow-up questionnaire and were asked to briefly meet one of the researchers in Amsterdam or Utrecht for handing over the ActiGraph and Fitbit devices. Participants who were not able to meet the researchers in person returned the ActiGraph and Fitbit by mail.

Participants (N_baseline_=13 [Active2Gether-Full=2, Active2Gether-Light=2, and Fitbit=9; mean delay of 7.5 days after end of previous measurement] and N_postintervention_=14 [Active2Gether-Full=0, Active2Gether-Light=3, and Fitbi*t* =11; mean delay of 24.4 days]) with insufficient ActiGraph data were asked to wear the accelerometer for another week. After completing the postintervention follow-up assessment and returning the devices, the participants received a voucher of €20 as an incentive for participating and an additional €5 for each participant they brought into the study, ranging from 0 to 15 additional euros.

### Measurements

#### Physical Activity

PA was assessed using 2 different assessment methods. The ActiGraph accelerometer was used to objectively measure the levels of PA to assess intervention effects. The Fitbit One also assesses PA objectively and was primarily used to allow participants to (self-)monitor their PA behavior, but the data were also used to explore possible intervention effects and to examine the levels of engagement.

Baseline and postintervention follow-up measurements were conducted using the ActiGraph GT3X+ (N=8) and ActiGraph wGT3XBT (N=32; ActiGraph Inc), and data were downloaded with the software ActiLife version 6.10.4. The ActiGraph GT3X has moderate validity and high reliability and is commonly used to assess PA in daily life [22-24]. The ActiGraph is a triaxial accelerometer that can convert accelerations to step counts. The sampling rate was set at 100 Hz, and afterward, data were aggregated to 1-min epochs. Participants were instructed to wear the accelerometer on the right hip using an elastic belt for 7 consecutive days during waking hours. Furthermore, they were instructed to remove the accelerometer during water activities and sleep. The accelerometer was set up with the specific information—gender, age, height, and weight—of the participant. Participants received a daily email containing a link to an online form asking to fill in the wear time of the day before.

Choi’s definitions and the *physical activity* R package were used to identify nonwear time (eg, periods of consecutive strings of 0s for at least 90 min; the time window for detecting and handling artifactual movement was set the default at 2 min). Interruptions up to 100 counts per minute within the string of 0s were filtered out [25].

Troiano’s definitions [26] were used to calculate the time spent per activity level using data from the three axes—vector magnitude score—of the ActiGraph; sedentary (<100 counts per minute), light (100-2019 counts per minute), moderate (2020-5998 counts per minute), vigorous (≥5999 counts per minute), and moderate-to-vigorous (≥2020 counts per minute) physical activities. To adjust for wear time, weekly minutes of MVPA—the sum of all minutes spent in MVPA during the assessment week—was divided by wear time, resulting in an average number of MVPA per day during the assessment week.

Participants were asked to wear a Fitbit One during 12 weeks to (self-)monitor their PA behavior. The Fitbit One (Fitbit Inc, San Francisco, California) tracker is a lightweight triaxial accelerometer with a built-in altitude monitor [21]. The Fitbit One assesses the step activity, active minutes, number of floors ascended, distance walked, and number of calories burned. The Fitbit One can be considered a valid device to assess daily step activity and step activity by using smaller time epochs and thus can be used for real-time minute-by-minute self-monitoring, although an overestimation of 677 steps per day by the Fitbit should be taken into account [27-30]. As there is no algorithm to define nonwear time for the Fitbit data, daily steps less than 1000 were treated as nonwear time [31-33]. Thus, only days with 1000 steps or more were included when Fitbit data were used to assess intervention effects and the levels of engagement.

#### Behavioral Determinants

Behavioral determinants that were addressed in the intervention were assessed with an online questionnaire at baseline, 6-week follow-up, and postintervention follow-up. Questionnaires that were used to assess the behavioral determinants were mainly based on existing and previously validated questionnaires.

#### Outcome Expectations

PA outcome expectations were assessed with 6 items using a 4-point Likert scale (1 [*I do not agree at all*] to 4 [*I totally agree*]). The statements captured expected outcome of PA with respect to health, appearance, weight, feeling fit, relaxation, and stress relief [34]. A sum score (range 6-24) was computed for each time point.

#### Self-Efficacy

Self-efficacy for PA was assessed with 13 items using a 5-point Likert scale (1 [*I know I can’t do it*] to 5 [*I am sure I can do it*]). The questionnaire was developed by Sallis et al [35] and translated into Dutch and used by Van Sluijs et al [36]. A sum score (range 13-65) was computed for each time point.

#### Barriers

Barriers for sports participation (N=12), active transport (N=7), and taking the stairs (N=4) were assessed using a 5-point Likert scale (1 [*never*] to 5 [*Very often*]) [34,37]. The list of barriers that was assessed was based on an existing questionnaire and previous focus group discussions with the target population [38]. A sum score was computed, summing the mean values of the three types of barriers—barriers for sports participation, active transport, and taking the stairs—(range 3-15) for each time point.

#### Intention

Intentions were assessed with three items using a 5-point Likert scale (1 [*very certainly not*] to 5 [*very certainly yes*]). Questions assessed the intentions to be physically active within the next week/month/6 months [34,37]. For the analysis, intentions to be physically active within the next month and the next 6 months were used.

#### Social Norm

Injunctive and descriptive social norms were assessed, where injunctive norms refer to the perceptions of what others think you are supposed to do, and descriptive norms refer to the perceptions of what others do [39]. Injunctive social norm was assessed with three items stated as “My sibling(s)/fellow students/friends think that I should be sufficiently physically active.” A 6-point Likert scale (1 [*I do not agree at all*] to 5 [*I totally agree*] and 6 [*not applicable*]) was used, and *not applicable* was coded as missing variables. A sum score (range 3-15) was computed for each time point.

Descriptive social norm was assessed with four items stated as “How often are your friends/fellow students/parents/siblings physically active?” A 6-point Likert scale (1 [*never*] to 5 [*very often*] and 6 [*not applicable*]) was used, and *not applicable* was coded as missing variables. A sum score (range 4-20) was computed for each time point.

#### Self-Regulation Skills

Self-regulation skills were assessed with seven items assessing exercise planning and scheduling and how the user keeps track of his/her activity and self-determined goals [40]. A 5-point Likert scale (1 [*never*] to 5 [*very often*]) was used. A sum score (range 7-35) was computed for each time point.

#### Satisfaction

Satisfaction was assessed using one item stating, “How satisfied are you with respect to how physically active you are on a scale from 0 to 10?”

#### Long-Term Goals

Satisfaction was assessed using one item stating, “How motivated are you to be (more) physically active on a scale from 0 to 10?”

#### Engagement and Usability

Engagement with the intervention was assessed using a number of coaching messages—only for the Active2Gether-Full condition—and Fitbit usage. As all participants were asked to wear the Fitbit during the intervention, we used the number of valid days the Fitbit was worn during 12 weeks (ie, 84 days).

A purpose-designed feedback questionnaire was used to examine the usability of the intervention. Users’ previous experiences with apps or activity trackers, self-reported usage of the Active2Gether app, and several aspects of user satisfaction—including encountering technical problems with the Active2Gether or Fitbit app—were assessed at postintervention follow-up.

Previous experiences with apps were assessed with a single question (“Did you have previous experience with PA apps prior to the current study?”) with three response options (“Yes, I use a PA app”; “Yes, I used to use a PA app, but now I don’t”; and “No, I have no previous experience with PA apps”). For the analyses, the variable was dichotomized (“Yes, have previous experiences” and “No, I don’t have any previous experience”).

Previous experiences with activity trackers were assessed with a single question (“Did you have previous experience with activity trackers prior to the current study?”) with three response options (“‘Yes, I use an activity tracker”; “Yes, I used to make use of an activity tracker, but now I don’t”; and “No, I have no previous experience with activity trackers”). For the analyses, the variable was dichotomized (“Yes, have previous experiences” and “No, I don’t have any previous experience”).

Usage of the Active2Gether app was assessed for the 2 Active2Gether conditions using a single question (“How often did you use the Active2Gether app?”), with an 8-point Likert scale (1 [*multiple times per day*] to 8 [*never*]). For the analyses, the variable was dichotomized (*multiple times per day*, *once per day*, and *multiple times per week* were coded as 1, whereas the options *once per week*, *multiple times per month*, *once per month*, *rarely*, and *never* were coded as 0).

Participants were asked how satisfied they were with the app they used (either 1 of the 2 versions of the Active2Gether app or the Fitbit app). A 7-point Likert scale was used to assess the level of agreement with the statement, “I am pleased with the app” (1 [*I do not agree at all*] to 7 [*I completely agree*]). For the analyses, the variable was categorized (*I do not agree at all* and *disagree* were coded as 1, *neutral* was coded as 2, and *I somewhat agree* and *I completely agree* were coded as 3).

Participants were asked whether they experienced technical problems with the app they used by asking the level of agreement with the statement, “I experienced technical problems with the app” on a 7-point Likert scale (1 [*I do not agree at all*] to 7 [*I completely agree*]). For the analyses, the variable was categorized (*I do not agree at all* and *disagree* were coded as 1, *neutral* was coded as 2, and *I somewhat agree* and *I completely agree* were coded as 3).

#### Demographics

Information on age, gender, and type of smartphone (iPhone/Android phone) was collected at registration through the Active2Gether website. Data on height (self-report), weight (self-report), and student status (yes/no) were requested at baseline during the intake session. Height and weight were used to calculate the body mass index (BMI).

### Sample Size

We used the G*Power software [41] and calculated the required sample size for a design with three groups (*F* test and analysis of variance [ANOVA]). As input, we used an effect size of 0.25, which is considered a medium effect size, an alpha of 5%, and a power of 80%. On the basis of these considerations, approximately 53 participants per group were required. Therefore, we aimed to include 159-200 participants, taking into account dropout and missing data.

### Statistical Analyses

#### Intervention Effects

Primary outcome variables were levels of PA at postintervention follow-up (ie, mean minutes of MVPA per day and mean steps per day), as measured by the ActiGraph. Secondary outcome variables were scores of behavioral determinants (ie, outcome expectations, self-efficacy, barriers, social norm, intentions, self-regulation skills, satisfaction, and long-term goals) at postintervention follow-up. Descriptive analyses were conducted for all variables—means and SDs (continuous variables) or frequencies and proportions (categorical variables). Chi-square tests (categorical variables) and one-way ANOVAs (continuous variables) were conducted to test for differences between groups at baseline.

For the analyses, the two intervention groups—the Active2Gether-Full and Active2Gether-Light conditions—were compared against a publicly available app, that is, the Fitbit app. This comparison will provide information on the effectiveness of the Active2Gether conditions compared with an existing *usual care* app. In addition, this design gives us the opportunity to compare the two Active2Gether conditions. As the difference between these two conditions is the inclusion or absence of the coaching, this comparison will provide information on the efficacy of the coaching part of the Active2Gether app. As participants with an iPhone were automatically assigned to the Fitbit condition and could not be randomly assigned to one of the two Active2Gether conditions, additional analyses were conducted to test for differences in intervention effects between the two Active2Gether conditions only. Furthermore, there were large differences in the duration of time between the start of the intervention and the postintervention follow-up measurements (ie, between 12 and 24 weeks). Thus, to examine the intervention effects at exactly 12-week follow-up, additional analyses were conducted using the Fitbit data (ie, step activity) instead of the ActiGraph data and the Fitbit data from the baseline week and 12-week follow-up were used.

For all analyses, regression techniques (linear and logistic) were used to examine the intervention effects. For this purpose, the assumptions were checked, and when necessary, variables were dichotomized.

In a first step, analyses were conducted to examine the efficacy of the intervention to increase weekly minutes of MVPA and weekly number of steps at postintervention follow-up. Associations were analyzed using linear regression analyses with the intervention conditions entered as dummy variables—the Fitbit condition was coded as the reference group—adjusting for baseline PA (ie, minutes of MVPA or number of steps) and time between baseline and postintervention follow-up. In a second step, analyses were conducted to examine the efficacy of the intervention to improve relevant behavioral determinants at postintervention follow-up. Linear regression analyses with the different determinants as dependent variables, while adjusting for baseline scores and time between baseline and postintervention measurements, were used. For dichotomous determinant variables (intentions and satisfaction), logistic regression analyses were conducted. These variables were dichotomized, as the residuals from the linear regression analyses when using the continuous variables were not normally distributed. All analyses were checked for outliers (≥3 SDs of the residuals), and when necessary, sensitivity analyses were conducted without outliers. The final analyses were conducted without outliers. A total of four models were run for each outcome variable (ie, levels of PA and scores of behavioral determinants): (0) a minimal adjusted model (only adjusted for baseline values and time between baseline and postintervention measurements), (1) a model additionally adjusted for BMI, (2) a model additionally adjusted for student status, (3) BMI models—(a) a model additionally adjusted for BMI and student status (for the intervention effects on PA only) and (b) a model adjusted for BMI and meeting the PA guidelines (for the intervention effects on behavioral determinants only). Owing to the small sample size, no further potential confounders were added to the final model.

#### Levels of Engagement and Usability

Exploratory analyses were conducted to evaluate how the users rated various aspects of the app they had used.

Descriptive statistics were provided for previous experiences with apps or activity trackers, usage of the Active2Gether app, satisfaction with the Active2Gether or Fitbit app, and encountering technical problems. Chi-square tests were used to examine differences in these variables between the groups.

#### Nonresponse Analyses

Nonresponse analyses were conducted to examine differences among those who had no PA data (assessed with the ActiGraph) for baseline and postintervention follow-up, those who only had baseline PA data, and those who had valid data at both baseline and postintervention follow-up. No significant differences between the groups were found with respect to age, BMI, student status, and all secondary outcome variables.

All analyses were conducted in STATA 14 (StataCorp, College Station, Texas).

## Results

### Baseline Characteristics

The baseline characteristics have been described in Table 1. A total of 104 participants (83 women) attended the intake session and completed the baseline questionnaire, and 98 participants had valid PA data for the baseline week. Figure 1 shows a flow diagram of the participants who dropped out, including reasons for dropping out. On average, participants were aged 23.4 years and had a BMI of 22.8 kg/m^2^; 69.2% (72/104) were students, 79.8% (83/104) were women, and 31.7% (33/104) had previous experiences with PA apps. At baseline, participants were, on average, moderately to vigorously active for 267.7 min per week. No significant differences between the Active2Gether conditions and Fitbit condition were found for the baseline characteristics.

**Table 1:** Baseline characteristics of participants in the Active2Gether-Full, Active2Gether-Light, and Fitbit conditions.

Characteristics	Overall	Active2Gether-Full	Active2Gether-Light	Fitbit	*P* value^a^
Participants, n (%)	104 (100)	28 (26.9)	27 (26.0)	49 (47.1)	N/A^b^
Female, n (%)	83 (79.8)	21 (75.0)	23 (85.2)	39 (79.6)	.96
Age (years), mean (SD)	23.4 (3.0)	23.7 (3.2)	22.8 (2.8)	23.5 (3.1)	.46
Body mass index (kg/m^2^), mean (SD)	22.8 (3.4)	23.8 (3.7)	22.6 (3.3)	22.3 (3.3)	.77
Student, n (%)	72 (69.2)	17 (60.7)	22 (81.5)	33 (67.3)	.69
Android phone, n (%)	57 (54.8)	28 (100)	27 (100)	3 (6.1)	<.001
Previous experience with physical activity apps (yes), n (%)	33 (31.7)	8 (28.6)	7 (25.9)	18 (36.7)	.46
Minutes of moderate-to-vigorous physical activity per week^c^, mean (SD)	267.7 (163.8)	234.9 (107.4)	258.8 (202.2)	293.1 (168.5)	.15
Step count using ActiGraph^c^, mean (SD)	8177.6 (3272.0)	7519.3 (2884.3)	7847.8 (3546.6)	8770.4 (3307.5)	.10
Step count using Fitbit^c^, mean (SD)	9008.9 (3722.8)	8179.9 (2415.9)	9190.7 (4610.6)	9535.5 (3878.0)	.30
Wear time for ActiGraph (minutes/day), mean (SD)	861.9 (61.3)	861.3 (50.5)	865.0 (58.8)	860.5 (69.6)	.84
Time between baseline and postintervention follow-up (days), mean (SD)^d^	103.4 (19.5)	106.5 (23.9)	109.0 (21.6)	97.7 (12.6)	.56

^a^Pearson Chi-square test with *P* value for frequencies and one-way analysis of variance for means for differences between Active2Gether-Full and Active2Gether-Light and Fitbit conditions.

^b^N/A: not applicable

^c^Baseline minutes of moderate-to-vigorous physical activity; number of steps and wear time were summed for the week and divided by the number of valid days to adjust for wear time.

^d^Intervention duration is the number of days between the start of the baseline assessment (day 1) and the last day of the postintervention follow-up assessment.

### Intervention Effects on Physical Activity

PA data (assessed with the ActiGraph) for baseline and postintervention follow-up were available for 88 participants (N_Active2Gether-Full_=25, N_Active2Gether-Light_=25, and N_Fitbit_=38). Table 2 shows the means and SDs for the outcome measurements for baseline and postintervention follow-up.

All results of the intervention effect on PA are discussed based on model 3a (adjustment for baseline PA, intervention duration, BMI, and student status).

Regression analyses showed no significant intervention effects of the Active2Gether-Full and Active2Gether-Light conditions on levels of PA (minutes of MVPA and steps) compared with the Fitbit condition. Effect sizes were small for average minutes of MVPA per day and smallest for the Active2Gether-Full condition (B=1.2; 95% CI −8.7 to 11.1). Thus, the Active2Gether-Full condition reported, on average, 1.2 min of MVPA per day more compared with the Fitbit condition. Table 3 shows the results of the regression analyses.

Additional regression analyses using the ActiGraph data showed a group difference of 2.8 min (95% CI −12.2 to 6.7) of MVPA per day between the Active2Gether-Full and Active2Gether-Light conditions in favor of the Active2Gether-Light condition (Multimedia Appendix 1). The same regression analyses, but using the Fitbit data at baseline and 12-week follow-up instead, showed a group difference of 533.51 steps per day (95% CI −2334.4 to 1267.4) between the Active2Gether-Full and Active2Gether-Light conditions in favor of the Active2Gether-Light condition (Multimedia Appendix 2).

**Table 2:** Characteristics at baseline (T1), 6-week follow-up (T2), and postintervention follow-up (T3).

Characteristics		Active2Gether-Full, mean (SD)			Active2Gether-Light, mean (SD)				Fitbit, mean (SD)			
		T1	T2	T3		T1	T2	T3		T1	T2	T3
**Physical activity measures**												
	Minutes of moderate-to-vigorous physical activity/day ActiGraph^a^	35.2 (15.3)	N/A^b^	39.7 (17.5)		38.6 (28.1)	N/A	42.1 (20.5)		43.5 (23.5)	N/A	44.8 (29.5)
	Steps/day ActiGraph^a^	7519.3 (2884.3)	N/A	7681.5 (2463.8)		7847.8 (3546.6)	N/A	8366.0 (2637.0)		8770.4 (3307.5)	N/A	9367.6 (4537.3)
	Steps/day Fitbit^c^	8179.9 (2415.9)	N/A	9392.8 (3275.7)		9190.7 (4610.6)	N/A	9567.1 (3152.2)		9535.5 (3878.0)	N/A	9968.1 (4506.2)
**Behavioral determinants (range of sum score)**												
	Self-efficacy (13-65)	42.4 (7.6)	41.8 (7.3)	42.8 (7.6)		42.5 (8.2)	40.9 (9.4)	42.0 (8.3)		44.5 (6.2)	45.4 (7.8)	44.7 (7.4)
	Outcome expectation (6-24)	20.3 (2.3)	19.7 (3.1)	19.8 (3.1)		20.4 (2.4)	19.3 (3.1)	19.5 (2.8)		20.4 (2.4)	20.5 (2.6)	19.8 (2.7)
	Social norm, injunctive (3-15)	10.7 (2.3)	10.9 (3.0)	10.3 (3.0)		9.9 (2.7)	10.2 (2.4)	9.7 (3.6)		9.9 (2.6)	9.8 (3.0)	10.8 (2.8)
	Social norm, descriptive (4-20)	14.6 (2.7)	14.9 (2.3)	14.8 (2.7)		13.4 (2.8)	13.2 (3.5)	13.2 (2.9)		13.9 (2.6)	13.8 (2.5)	13.2 (2.7)
	Intention in 1 month (1-5)	4.1 (0.7)	3.6 (1.0)	3.5 (1.0)		3.7 (1.1)	3.1 (1.3)	3.4 (1.2)		3.9 (0.8)	3.4 (1.0)	3.3 (1.0)
	Intention in 6 months (1-5)	4.4 (0.8)	3.8 (0.8)	3.9 (0.9)		4.1 (0.9)	3.7 (1.1)	3.7 (0.9)		4.2 (0.7)	3.6 (0.9)	3.5 (1.0)
	Barriers (3-15)	8.4 (1.7)	8.3 (1.9)	8.2 (1.9)		7.9 (1.5)	7.8 (1.4)	7.9 (1.5)		7.7 (1.7)	7.7 (1.9)	7.7 (1.7)
	Self-regulation skills (5-25)	18.8 (4.3)	19.4 (3.2)	19.3 (3.8)		19.0 (5.5)	20.1 (5.5)	19.3 (5.0)		20.9 (4.6)	21.0 (4.4)	20.8 (4.4)
	Satisfaction (0-10)	5.5 (1.8)	6.0 (1.7)	5.9 (2.0)		5.5 (1.8)	5.5 (1.8)	5.7 (1.7)		6.0 (1.7)	6.2 (1.9)	6.3 (1.9)

^a^Minutes of moderate-to-vigorous physical activity and number of steps per day assessed with ActiGraph.

^b^Not applicable.

^c^Number of steps per day assessed with Fitbit.

**Table 3:** Results of the regression analyses to evaluate the intervention effects of the Active2Gether-Full and Active2Gether-Light condition on levels of physical activity at postintervention follow-up compared with the Fitbit condition.

Parameter^a^ and condition		Model 0^b^			Model 1^c^: BMI^d^			Model 2^e^: student			Model 3a^f^: BMI-student	
		B	95% CI	B		95% CI	B		95% CI	B		95% CI
**Average minutes of moderate-to-vigorous physical activity per day**												
	Fitbit	Reference	Reference	Reference		Reference	Reference		Reference	Reference		Reference
	Active2Gether-Full	0.82	−8.82 to 10.46	1.16		−8.73 to 11.04	0.92		−8.70 to 10.54	1.20		−8.66 to 11.07
	Active2Gether-Light	1.99	−7.56 to 11.55	2.14		−7.51 to 11.78	3.00		−6.68 to 12.67	3.10		−6.66 to 12.87
**Average number of steps per day**												
	Fitbit	Reference	Reference	Reference		Reference	Reference		Reference	Reference		Reference
	Active2Gether-Full	−577.42	−1913.68 to 758.85	−387.88		−1742.21 to 966.44	−575.76		−1918.33 to 766.82	−388.95		−1750.20 to 972.31
	Active2Gether-Light	−128.54	−1447.23 to 1190.16	−45.56		−1361.36 to 1270.24	−70.37		−1413.70 to 1272.96	5.21		−1334.82 to 1345.25

^a^Linear regression analyses are presented with regression coefficient (B) and 95% CI, and all analyses were adjusted for levels of physical activity at baseline and time between baseline and postintervention follow-up.

^b^Model 0: y=B_0_+B_1_×physical activity at postintervention+B_2_×physical activity at baseline+B_3_×time until postintervention follow-up (days).

^c^Model 1: Model 0+B_4_×BMI (kg/m^2^).

^d^BMI: body mass index.

^e^Model 2: Model 0+B_4_×student (yes/no).

^f^Model 3: Model 0+B_4_×BMI (kg/m^2^)+B_5_×student (yes/no).

### Intervention Effects on Behavioral Determinants

Survey data for baseline and 12-week follow-up were available for 92 participants (N_Active2Gether-Full_=24, N_Active2Gether-Light_=23, and N_Fitbit_=45). Table 2 shows the mean and SD for behavioral determinant scores for baseline, 6-week follow-up, and postintervention follow-up.

Linear and logistic regression analyses for the intervention effects on the sum score of the behavioral determinants at postintervention follow-up showed no significant intervention effects of the Active2Gether-Full and Active2Gether-Light conditions compared with the Fitbit condition. For all analyses, small effect sizes were found, except for intentions to be physically active within 6 months (Model 3b: odds ratio [OR]_Active2Gether-Full_=2.13, 95% CI 0.59-7.75; OR_Active2Gether-Light_ 3.57, 95% CI 0.93-13.72). Thus, participants in the Active2Gether-Full condition have an OR of 2.13 to have high intentions to be physically active at 6 months compared with the Fitbit condition, whereas participants in the Active2Gether-Light condition have an OR of 3.57. Table 4 shows the results of the regression analyses. Additional analyses showed that participants in the Active2Gether-Full condition have an OR of 0.72 (95% CI 0.15-3.51) to have high intentions to be physically active at 6 months compared with the Active2Gether-Light condition (Multimedia Appendix 3).

**Table 4:** Results of the linear and logistic regression analyses to evaluate the intervention effects of the Active2Gether-Full and Active2Gether-Light conditions on behavioral determinants at postintervention follow-up compared with the Fitbit condition.

Outcome measurement^a^ and condition		Model 0^b^	Model 1^c^: BMI^d^	Model 2^e^: student	Model 3b^f^: BMI-PA
**Self-efficacy, B (95% CI)^g^**					
	Fitbit	Reference	Reference	Reference	Reference
	Active2Gether-Full	0.03 (−2.88 to 2.94)	0.74 (−2.15 to 3.63)	0.14 (−2.73 to 3.00)	0.62 (−2.24 to 3.49)
	Active2Gether-Light	−1.52 (−4.40 to 1.36)	−1.28 (−4.08 to 1.52)	−0.92 (−3.81 to 1.98)	−1.54 (−4.34 to 1.25)
**Outcome expectations, B (95% CI)^g^**					
	Fitbit	Reference	Reference	Reference	Reference
	Active2Gether-Full	0.44 (−0.59 to 1.47)	0.43 (−0.63 to 1.50)	0.40 (−0.61 to 1.41)	0.41 (−0.66 to 1.47)
	Active2Gether-Light	0.07 (−0.95 to 1.10)	0.07 (−0.97 to 1.11)	−0.12 (−1.15 to 0.91)	0.02 (−1.02 to 1.07)
**Social norm, descriptive, B (95% CI)^g^**					
	Fitbit	Reference	Reference	Reference	Reference
	Active2Gether-Full	1.18 (0.15 to 2.20)	1.11 (0.05 to 2.16)	1.26 (0.24 to 2.29)	1.12 (0.08 to 2.16)
	Active2Gether-Light	−0.14 (−1.13 to 0.86)	−0.16 (−1.16 to 0.84)	0.02 (−1.00 to 1.03)	−0.06 (−1.05 to 0.94)
**Social norm, injunctive, B (95% CI)^g^**					
	Fitbit	Reference	Reference	Reference	Reference
	Active2Gether-Full	0.11 (−1.64 to 1.85)	0.27 (−1.53 to 2.06)	0.26 (−1.47 to 2.00)	0.03 (−1.73 to 1.80)
	Active2Gether-Light	−0.45 (−2.05 to 1.16)	−0.33 (−1.97 to 1.30)	−0.09 (−1.74 to 1.56)	−0.42 (−2.01 to 1.18)
**Intention in 1 month, OR (95% CI)^h^**					
	Fitbit	Reference	Reference	Reference	Reference
	Active2Gether-Full	1.01 (0.33 to 3.06)	0.91 (0.29 to 2.85)	1.01 (0.33 to 3.06)	0.92 (0.29 to 2.90)
	Active2Gether-Light	1.37 (0.45 to 4.13)	1.32 (−0.43 to 4.02)	1.38 (0.45 to 4.24)	1.39 (0.45 to 4.33)
**Intention in 6 months, OR (95% CI)^h^**					
	Fitbit	Reference	Reference	Reference	Reference
	Active2Gether-Full	2.66 (0.77 to 9.23)	2.08 (0.58 to 7.50)	2.66 (0.77 to 9.22)	2.13 (0.59 to 7.75)
	Active2Gether-Light	3.28 (0.92 to 11.76)	3.30 (0.88 to 12.38)	3.24 (0.87 to 12.04)	3.57 (0.93 to 13.72)
**Barriers, B (95% CI)^g^**					
	Fitbit	Reference	Reference	Reference	Reference
	Active2Gether-Full	−0.01 (−0.60 to 0.58)	−0.20 (−0.77 to 0.37)	0.03 (−0.54 to 0.61)	−0.20 (−0.77 to 0.37)
	Active2Gether-Light	0.06 (−0.53 to 0.64)	−0.00 (−0.55 to 0.54)	0.16 (−0.41 to 0.73)	0.00 (−0.56 to 0.56)
**Self-regulation skills, B (95% CI)^g^**					
	Fitbit	Reference	Reference	Reference	Reference
	Active2Gether-Full	0.78 (−0.94 to 2.50)	0.80 (−0.96 to 2.56)	0.82 (−0.90 to 2.54)	0.83 (−0.94 to 2.60)
	Active2Gether-Light	0.01 (−1.68 to 1.69)	0.02 (−1.69 to 1.72)	0.20 (−1.53 to 1.93)	0.09 (−1.63 to 1.80)
**Satisfaction, OR (95% CI)^h^**					
	Fitbit	Reference	Reference	Reference	Reference
	Active2Gether-Full	0.69 (0.19 to 2.52)	0.88 (0.23 to 3.40)	0.81 (0.22 to 3.03)	0.88 (0.23 to 3.42)
	Active2Gether-Light	0.49 (0.14 to 1.75)	0.51 (0.14 to 1.87)	0.65 (0.18 to 2.30)	0.50 (0.13 to 1.85)

^a^All analyses were adjusted for baseline scores of the determinant and time between baseline and postintervention follow-up.

^b^Model 0: y=B_0_+B_1_×determinant at postintervention+B_2_×determinant at baseline+B_3_×time until postintervention follow-up (days).

^c^Model 1: Model 0+B_4_×BMI (kg/m^2^).

^d^BMI: body mass index.

^e^Model 2: Model 0+B_4_×student (yes/no).

^f^Model 3: Model 0+B_4_×student (yes/no)+B_5_×meeting physical activity guidelines at baseline (yes/no).

^g^Linear regression analyses are presented with regression coefficient (B; 95% CI).

^h^Logistic regression analyses with odds ratio (OR; 95% CI).

### Levels of Engagement and Usability

For the Active2Gether-Full condition, 1429 messages were derived, 1381 messages (ie, 97% (1381/1429) of the messages) were sent, and 1324 messages were successfully received. For 5 of the 24 users, a derived message was not sent at some point, which could indicate that the app was removed before the end of the study. For nine users, a sent message was not received by phone, and one user did not receive any messages at all.

**Figure 2:**
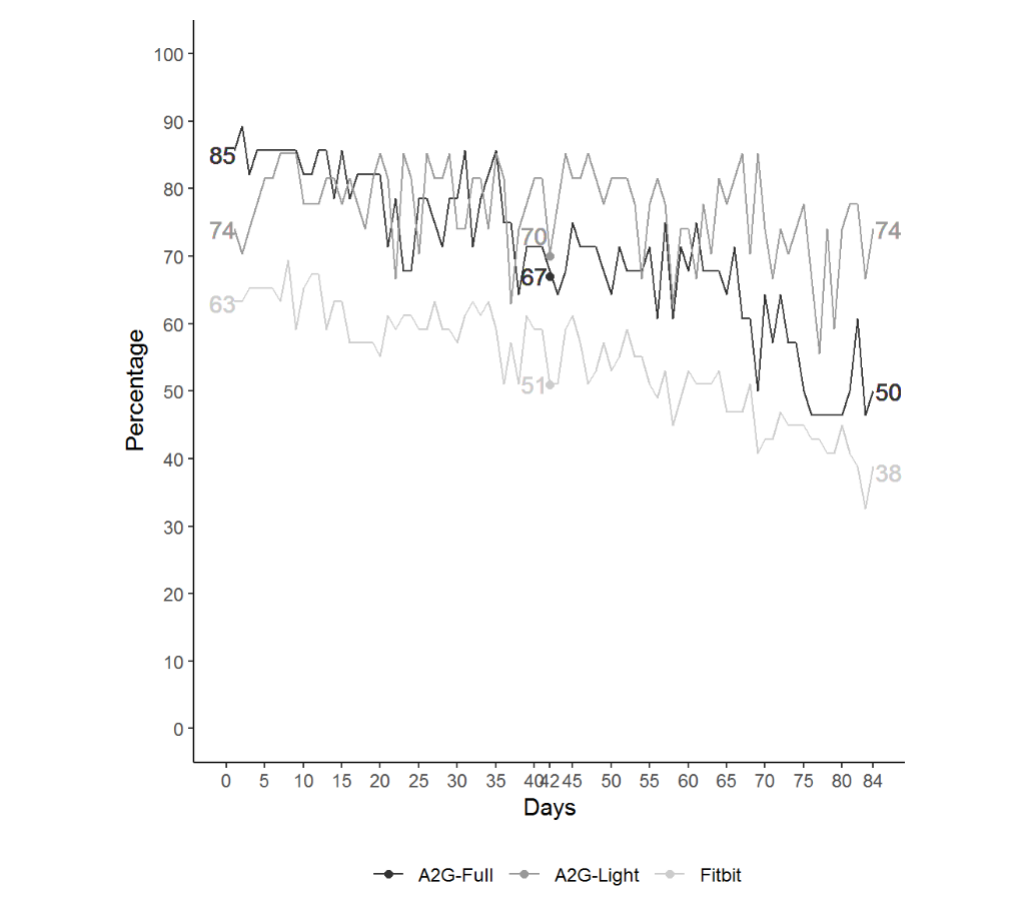
Fitbit usage in the participants who used the Fitbit throughout the intervention period of 12 weeks. This figure shows the proportions of participants who recorded step activity (>1000 steps per day) assessed with the Fitbit for the three conditions: Active2Gether-Full (A2G-Full), Active2Gether-Light (A2G-Light), and Fitbit.

For participants in the Active2Gether-Full and Fitbit conditions, a decrease was observed (from day 1 to day 84 of the intervention) in the number of participants who recorded valid step activity (>1000 steps per day) assessed with the Fitbit. At the 6-week follow-up (ie, after 42 days), 68% (19/28) of the Active2Gether-Full condition, 70% (19/27) of the Active2Gether-Light condition, and 51% (25/49) of the Fitbit condition were still using the Fitbit. At 12-week follow-up (ie, after 84 days), 50% (14/28) of the Active2Gether-Full condition, 74% of the Active2Gether-Light condition, and 38% (19/49) of the Fitbit condition were still using the Fitbit. Figure 2 shows the number of participants who logged step activity per intervention condition, and a steeper decrease was seen for the Fitbit condition relative to the 2 Active2Gether conditions.

**Figure 3:**
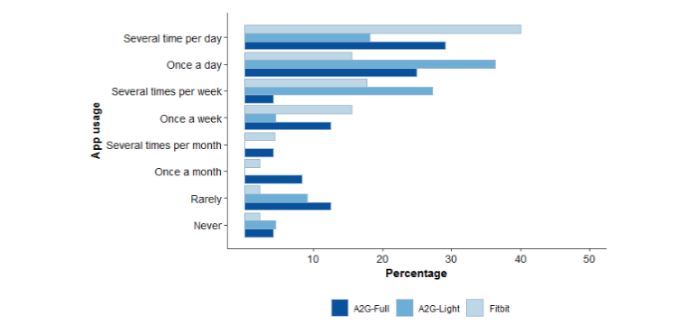
Frequency plot for app usage during the intervention period per intervention group. A2G: Active2Gether. App usage scores: 1, never; 2, rarely; 3, once a month; 4, several times per month; 5, once per week; 6, several times per week; 7, once a day; and 8, several times per day.

The majority (58% (14/28) and 82% (18/22)) of the participants in the Active2Gether-Full and Active2Gether-Light conditions, respectively, reported that they used the app at least several times per week or more frequently (Figure 3); for the Fitbit condition, this value was 73% (33/45). Significant differences were found in how satisfied the participants were with the app they used during the intervention. The majority of participants in the two Active2Gether conditions were not satisfied with the app (Active2Gether-Full=67% (16/24) and Active2Gether-Ligh*t* =64% (14/22)), whereas 22% of the participants in the Fitbit condition were not satisfied with the Fitbit app. More participants in the two Active2Gether conditions (Active2Gether-Full=54% (13/24) and Active2Gether-Ligh*t* =45%) experienced technical problems with the app compared with the Fitbit condition (23% (10/44)). Table 5 shows the scores on the user evaluations.

A more detailed evaluation of the user experience of the Active2Gether intervention can be found elsewhere [42].

**Table 5:** User engagement and usability of the Active2Gether-Full, Active2Gether-Light, and Fitbit apps assessed at the postintervention follow-up.

Measurement of engagement and usability		Overall	Active2Gether-Full	Active2Gether-Light	Fitbit	*P* value^a^
Fitbit usage, median percentage of days used^b^ (range)		88 (0-100)	86(10-100)	95 (4-100)	84 (0-100)	.13
**Previous experience with physical activity apps^c^** **, n (%)**						
	Yes	33 (36)	8 (33)	7 (32)	18 (40)	.47
**Previous experience with activity trackers^c^, n (%)**						
	Yes	17 (19)	6 (25)	4 (18)	7 (16)	.45
**Satisfied with the Active2Gether or Fitbit app^d^, n (%)**						
	Yes	36 (40)	5 (21)	5 (23)	26 (58)	<.001
	Neutral	15 (16)	3 (13)	3 (14)	9 (20)	<.001
	No	40 (44)	16 (67)	14 (64)	10 (22)	<.001
**Experienced technical problems with the app^d^, n (%)**						
	Yes	33 (37)	13 (54)	10 (45)	10 (23)	.009
	Neutral	3 (3)	0 (0)	0 (0)	3 (7)	.009
	No	54 (60)	11(46)	12 (55)	31 (70)	.009
**App usage^e^, n (%)**						
	Often	76 (84)	14 (63)	18 (82)	33 (73)	.69

^a^*P* value is the result of a chi-square test between Active2Gether users (Full and Light version) versus Fitbit users.

^b^Percentage of days used=number of days the Fitbit was used (steps>1000)/84 days×100.

^c^The score was dichotomized: Yes=Yes, I’m currently using one and Yes, in the past and No=No, no experience.

^d^The score was categorized: Yes=agree, somewhat agree, and totally agree; Neutral=neutral; and No=completely disagree, somewhat disagree, and disagree.

^e^The score was dichotomized: rarely=never, rarely, once a month, multiple times per month, and once per week; and often=multiple times per week, once a day, and multiple times per day.

## Discussion

### Principal Findings

This study aimed to explore whether two versions of the Active2Gether app—a tailored app-based intervention to promote PA—appeared to be more effective in increasing the levels of PA among young adults than an existing self-monitoring app. The secondary aims of the study were to examine and explore whether the intervention was effective in changing the levels of relevant behavioral determinants of PA and how participants used and evaluated the app. No evidence for significant intervention effects on increased PA or more positive determinants of PA were found. Most Active2Gether app users used the app at least several times per week and were not satisfied with the app, and a substantial number of participants experienced technical problems.

This study was originally designed and planned as a randomized controlled trial (RCT) with 159 to 200 participants and a follow-up measurement for all participants at 12 weeks, that is, immediately after the envisioned intervention period. Owing to the practicalities and challenges encountered, the study conducted differed substantially from the original protocol. Despite these explicitly acknowledged suboptimal design and power, we wish to share our results with the scientific community to contribute to the further development of artificial intelligence–supported, individually tailored health behavior promoting interventions and to help avoid publication bias.

First, the number of participants was lower than that envisioned in the study protocol. Despite our efforts for participant recruitment, fewer people than expected were willing to participate because of a lack of interest, a lack of time, and the perceived burden for the participants. Owing to the smaller sample, the statistical power of the results was lower than that according to protocol.

In addition, participants were not assigned to the three conditions based on true randomization. One reason for this is that the two versions of the Active2Gether app (Active2Gether-Full and Active2Gether-Light) could not be made available for iPhone users; therefore, iPhone users were automatically assigned to the Fitbit condition. In addition, the proportion of Android users who registered for the study was lower than expected; therefore, to maintain a balance between the three conditions, they were randomized over the two Active2Gether conditions only, rather than over all three conditions. Therefore, the study would ideally only include Android users, or the Active2Gether intervention should have been made available for iPhones as well.

Third, owing to the difficulties with recruiting participants from the target population, the inclusion of the participants was spread over 3 months. Consequently, some participants were included just at the end of the academic year and the beginning of the summer holidays. As a result, the 12-week follow-up measurements were due in the middle of their summer holiday for the majority of the participants. Therefore, the postintervention measures were delayed, and the time between the baseline and postintervention follow-up varied widely among the participants.

Finally, due to the malfunction of the PA assessment with the ActiGraph, the baseline measurement had to be redone for a number of participants. Therefore, the baseline measurement of PA for some participants took place during the intervention, rather than at the start.

Despite these major violations of the original study protocol, we want to discuss the results found in more detail, but this discussion should, of course, be read and interpreted while keeping these differences between the study designed and the one conducted in mind.

No statistically significant effects were found, and the effect sizes were small: Compared with the Active2Gether-Light condition, the Active2Gether-Full condition measured, on average, 2.76 min of MVPA less per day, accounting to 19.32 min of MVPA per week. In addition, based on Fitbit registrations, the Active2Gether-Light users took 533.51 more steps per day. Earlier evaluations of app-based interventions also reported mixed results, but the majority of the studies reported significant intervention effects relative to the control group. Those studies reported changes between −15.5% and 34.8% in PA in the intervention groups, of whom the majority evaluated the intervention effects at 8-week follow-up [43-46]. However, it should be noted that these studies differ with respect how they assess PA: One study used the ActiGraph [43], a pedometer to assess step activity [44], a validated questionnaire [40,43], and a built-in smartphone accelerometer to assess PA with an unknown validity [45]. Owing to the different assessment methods used in the different studies, it is difficult to compare the results. Furthermore, the participants in this study were already active and, on average, met the guidelines of 30 min MVPA per day, whereas the baseline PA levels in other studies were much lower. As it might be difficult to increase weekly levels of MVPA in an already active group, this might partially explain the lack of intervention effect.

The secondary aim of this study was to examine whether the Active2Gether-Full intervention effectively changes scores in behavioral determinants that were included in the theoretical framework. No significant intervention effects were seen in changes in scores, indicating that sending the tailored coaching messages did not lead to changes in the behavioral determinants. A meta-analysis reported significant higher effect sizes for self-efficacy and for PA in interventions for adults with obesity when prompt self-monitoring of behavioral outcome and plan social support/social change were included [47]. However, little is known about the effects of behavior change techniques on behavioral determinants for app-based interventions. Thus far, the only study examining the effects of the Fitbit app on social cognitive behavioral determinants showed no significant changes in behavioral determinants after 12 weeks [33]. Other studies using apps to change PA used of self-monitoring features, motivational messages, and prompts and offered challenges to increase the levels of PA as well but did not examine changes in behavioral determinants [43-45]. Therefore, it remains unclear whether these app-based interventions successfully changed the underlying and relevant behavioral determinants. Therefore, future research is needed to examine whether motivational messages, prompts, challenges, and social support features can be used to change behavioral determinants. For this, a more iterative assessment of the determinants during the intervention is needed, as performed in the Active2Gether intervention. Consequently, this knowledge will contribute to further tailoring and personalizing app-based interventions to increase levels of PA.

Although 96 participants (96/104, 92.3%) participated in the postintervention follow-up assessment, lower rates of engagement with the Fitbit were seen after 12 weeks, especially for the Fitbit condition. However, the overall engagement with the Fitbit was high (median 88% of the days). This is in line with the self-reported app usage: Most participants reported that they used the appointed app several times per week or more throughout the intervention. However, about only 21.2% (22/104) and 23.1% (24/104) of the participants in the Active2Gether-Full and Active2Gether-Light condition, respectively, were satisfied with the app, whereas 57.7% (60/104) of the participants were satisfied with the Fitbit app in the control condition. Those low scores might be related to the high rates of technical problems that the participants in the Active2Gether conditions encountered and the participants’ high expectations of an app. Moreover, it should be noted that the Fitbit used to monitor daily activity did not automatically synchronize with the Active2Gether apps. The participants in the 2 Active2Gether conditions needed to synchronize the Fitbit through the Fitbit app or Fitbit website. This additional step can be a burden for the users of the Active2Gether apps and might be more prone to technical errors. The Active2Gether-Full app sent the weekly questions and coaching messages via push messages, and the users could only access the app after reading the unread messages. Participants in the Active2Gether-Light condition only received daily or weekly questions via push messages. A more detailed evaluation of the participants’ satisfaction in the usability of the app is published elsewhere [42].

This study compared the Active2Gether conditions to the Fitbit app. The Fitbit app enables users to monitor their activity (eg, number of steps, floors climbed, and distance walked), monitor their sleep, and set activity goals. In addition, users have the possibility to log their weight, calorie, and fluid intake. Thus, compared with both the Active2Gether conditions, the Fitbit app enables tracking of various lifestyle components instead of only tracking activity levels. Fitbit sends its users weekly emails with a weekly summary of their progress and congratulations on earning badges. However, participants were asked to register using an Active2Gether email address so that they would not receive these emails in this study. In brief, the Fitbit app included behavior change techniques that were also embedded in the Active2Gether-Full condition.

To summarize, this study showed no significant intervention effects in changes in levels of PA and behavioral determinants compared with the active control groups. Because the study conducted differed substantially from the study designed, any attempt to explain these results should be done with utmost caution. First, the lack of effects found may be because of the lack of an internally valid research design: We had nonrandom allocation between the two Active2Gether conditions and the control *Fitbit* condition. In addition, the number of participants was smaller than we aimed for based on our power analysis, and there was a large variation in at the postintervention measurement. As the effect sizes were generally small, it is unlikely that the lack of sufficient power explains the lack of statistically significant differences between the conditions, although the differences between the baseline and postintervention assessments in minutes of MVPA were 12% to 15% in the Active2Gether conditions, which may be an indication that these Active2Gether interventions do warrant further research. The lack of effect might also be because of the lack of exposure to the interventions; a large majority of the participants did not make use of the app, as we assumed was needed to have sufficient influence and impact on determinants and behavior. Such lack of true exposure to mobile health and electronic health interventions has been found before [16], and a main focus in further research should be how exposure to and actual use of such interventions can be intensified. A research by Schoeppe et al [16] suggests that the effects of app-based interventions as part of more comprehensive, multicomponent programs that may also include other forms of health education of face-to-face counseling may be more likely to be effective.

### Strengths and Limitations

The main research design–related limitations of this study have already been described in the Introduction section of this paper and in the opening paragraphs of Discussion section: the lack of full randomization, the small sample size, the variation in timing of the postintervention measurement, and the fact that the baseline measurement of PA for some participants took place during intervention exposure. In addition, most participants were highly educated, female, and already more physically active than the population at large, which limits the external validity. Furthermore, about half of the participants in the Active2Gether conditions experienced technical problems with their app; however, only a few participants informed the researchers that they were having technical problems. Consequently, they might have stopped using the app without first requesting assistance with solving the problem.

Strengths of this study are the high completion rate for participants (92% (95/104)) and the fact that the experimental interventions were compared to Active2Gether with an existing app (the Fitbit app). Comparing the Active2Gether-Full app with the Active2Gether-Light version further provided information about whether sending tailored coaching messages on top of the monitoring and social comparison had an added effect on PA. Another strength was the use of the ActiGraph accelerometer—a valid and reliable accelerometer—to objectively assess baseline and postintervention follow-up PA and the use of existing questionnaires to assess the behavioral determinants. Further evaluation is needed to examine whether sending coaching messages resulted in changes in step activity throughout the study period.

### Suggestions for Future Research

This study was originally designed and planned as an RCT with baseline, 6-week, and 12-week evaluations. However, as app-based interventions are relatively new in health promotion and offer the possibility to provide highly personalized and just-in-time feedback, it is necessary to evaluate the long-term effects and what parts of the intervention are effective and what works for whom. RCTs provide information on the overall effects of the intervention, and they come with some challenges as well. First, choosing an appropriate control group: Compare the intervention to another intervention, no intervention, or a waitlist control group. Second, a truly controlled environment for a trial in real-life circumstances (ie, outside a behavior laboratory) is a challenge, as participants may use other apps or intervention activities to monitor and help them increase or maintain PA levels alongside the intervention to be tested in the study. On the other hand, such opportunities for using existing and available apps or other intervention activities are part of the real-life circumstances, and testing a new app in such circumstances will show if this app has effects additional to what is already available. Therefore, a future study should consider an RCT design with sufficient power, only after the app has been developed, and pilot a more agile developmental process.

### Conclusions

This study showed no statistically significant effect of the Active2Gether-Full condition compared with the Active2Gether-Light and Fitbit conditions. Future work is needed to increase the actual use of the apps to integrate the apps in a more comprehensive, multicomponent intervention and in a study with better internal validity.

## Appendix

Multimedia Appendix 1 Results of the linear regression analyses (regression coefficients [B] with 95% CI) for differences in physical activity at postintervention follow-up between Active2Gether-Full and Active2Gether-Light conditions assessed with ActiGraph.

Multimedia Appendix 2 Results of the linear regression analyses (regression coefficients [B] with 95% CI) for differences in step activity at postintervention follow-up using the Fitbit data (N=63).

Multimedia Appendix 3 Linear and logistic regression analyses for differences in behavioral determinants between Active2Gether-Full and Active2Gether-Light conditions at postintervention follow-up adjusted for baseline and time between baseline and postintervention follow-up.

## Abbreviations

ANOVA: analysis of variance
BMI: body mass index
MVPA: moderate-to-vigorous physical activity
PA: physical activity
RCT: randomized controlled trial
