# Supplementary material for: The Use and Effects of an App-Based Physical Activity Intervention “Active2Gether” in Young Adults: Quasi-Experimental Trial
Source: JMIR Form Res. 2020 Jan 21;4(1):e12538. doi: 10.2196/12538 (PMC7001048; doi:10.2196/12538)
Supplement: Multimedia Appendix 1 [file formative_v4i1e12538_app1.pdf]

Multimedia appendix 1 – Results of the linear regression analyses (regression coefficients (B) with 95% confidence intervals (95%CI)) for differences in physical activity at post-intervention follow-up between Active2Gether-Full and Active2Gether-Light assessed with the ActiGraph

	**Average minutes of moderate-vigorous physical activity per day**			
	**Model 0**<br>**B (95% CI)**	**Model 1: BMI**<br>**B (95% CI)**	**Model 2: Student**<br>**B (95% CI)**	**Model 3a: BMI-Student**<br>**B (95% CI)**
Active2Gether-Light	Reference	Reference	Reference	Reference
Active2Gether-Full	-1.90 [-10.84,7.05]	-2.34 [-11.51,6.83]	-2.41 [-11.66,6.84]	-2.76 [-12.20,6.69]
	**Average number of steps per day**			
Active2Gether-Light	Reference	Reference	Reference	Reference
Active2Gether-Full	-591.72 [-1875.72,692.29]	-549.39 [-1867.27,768.49]	-685.61 [-2011.45,640.23]	-641.71 [-1996.37,712.96]

Note. Linear regression analyses are presented with regression coefficient (B) [95% confidence interval (95%CI)], and all analyses were adjusted for levels of physical activity at baseline and time between baseline and post-intervention follow-up

Model 0: y= B_0 + B_1*Physical activity at post-intervention + B_2* Physical activity at baseline + B_3* Time until post-intervention follow-up (days)

Model 1: Model 0 + B_4*BMI (kg/m^2)

Model 2: Model 0 + B_4*Student (yes/no)

Model 3: Model 0 + B_4* BMI (kg/m^2) + B_5* Student (yes/no)
