# Supplementary material for: The Use and Effects of an App-Based Physical Activity Intervention “Active2Gether” in Young Adults: Quasi-Experimental Trial
Source: JMIR Form Res. 2020 Jan 21;4(1):e12538. doi: 10.2196/12538 (PMC7001048; doi:10.2196/12538)
Supplement: Multimedia Appendix 2 [file formative_v4i1e12538_app2.pdf]

Multimedia appendix 2 – Results of the linear regression analyses (regression coefficients (B) with 95% confidence intervals (95%CI)) for differences in step activity at post-intervention follow-up using the Fitbit data (N=64)

	**Average number of steps per day assessed with the Fitbit One for all three conditions**			
	**Model 0**	**Model 1: BMI**	**Model 2: Student**	**Model 3a: BMI-Student**
Fitbit	Reference	Reference	Reference	Reference
Active2Gether-Full	-827.90 [-2849.7,1193.9]	-804.41 [-2878.1,1269.3]	-820.43 [-2858.4,1217.6]	-787.52 [-2879.4,1304.3]
Active2Gether -Light	-1550.98 [-3421.4,319.5]	-1548.68 [-3436.0,338.6]	-1566.56 [-3453.5,320.4]	-1564.31 [-3468.2,339.5]
	**Average number of steps per day assessed with the Fitbit One for Active2Gether-*Full* versus Active2Gether-*Light***			
Active2Gether-Light	Reference	Reference	Reference	Reference
Active2Gether -Full	-516.59 [-2226.9,1193.7]	-519.13 [-2294.1,1255.8]	-531.64 [-2266.7,1203.4]	-533.51 [-2334.4,1267.4]

Note. Linear regression analyses are presented with regression coefficient (B) [95% confidence interval]. For the analyses, the Fitbit data was used for baseline (day 1-day 7) and 12 weeks follow-up (day 78-day 84)

Model 0: y= B_0 + B_1*Physical activity at post-intervention + B_2* Physical activity at baseline + B_3* Time until post-intervention follow-up (days)

Model 1: Model 0 + B_4*BMI (kg/m^2)

Model 2: Model 0 + B_4*Student (yes/no)

Model 3: Model 0 + B_4* BMI (kg/m^2) + B_5* Student (yes/no)
