# Supplementary material for: The Use and Effects of an App-Based Physical Activity Intervention “Active2Gether” in Young Adults: Quasi-Experimental Trial
Source: JMIR Form Res. 2020 Jan 21;4(1):e12538. doi: 10.2196/12538 (PMC7001048; doi:10.2196/12538)
Supplement: Multimedia Appendix 3 [file formative_v4i1e12538_app3.pdf]

Multimedia appendix 3 – Linear and logistic regression analyses for differences in behavioral determinants between Active2Gether-Full and Active2Gether-Light at post-intervention follow-up adjusted for baseline and time between baseline and post-intervention follow-up

Outcome measurement	Condition	Model 0	Model 1: BMI	Model 2: Student	Model 3b: BMI-PA
Self-efficacy (B [95% CI])	Active2Gether-Light	Reference	Reference	Reference	Reference
	Active2Gether-Full	1.55 [-1.79,4.89]	2.03 [-1.35,5.42]	1.08 [-2.38,4.53]	2.15 [-1.25,5.54]
Outcome expectations (B [95% CI])	Active2Gether-Light	Reference	Reference	Reference	Reference
	Active2Gether-Full	0.62 [-0.35,1.59]	0.57 [-0.44,1.58]	0.82 [-0.14,1.79]	0.58 [-0.43,1.59]
Social norm descriptive (B [95% CI])	Active2Gether-Light	Reference	Reference	Reference	Reference
	Active2Gether-Full	1.10 [0.16,2.04]	1.01 [0.03,1.98]	1.18 [0.22,2.13]	1.02 [0.03,2.01]
Social norm injunctive (B [95% CI])	Active2Gether-Light	Reference	Reference	Reference	Reference
	Active2Gether-Full	0.53 [-1.34,2.39]	0.48 [-1.45,2.41]	0.54 [-1.47,2.54]	0.30 [-1.67,2.26]
Intention 1 month (OR [95% CI])	Active2Gether-Light	Reference	Reference	Reference	Reference
	Active2Gether-Full	0.49 [0.13,1.79]	0.38 [0.09,1.49]	0.44 [0.12,1.66]	0.38[0.10,1.53]
Intention 6 months (OR [95% CI])	Active2Gether-Light	Reference	Reference	Reference	Reference
	Active2Gether-Full	0.89 [0.20,3.93]	0.77 [0.16,3.71]	0.72 [0.15,3.55]	0.72 [0.15,3.51]
Barriers (B [95% CI])	Active2Gether-Light	Reference	Reference	Reference	Reference
	Active2Gether-Full	-0.02 [-0.71,0.67]	-0.13 [-0.82,0.57]	-0.08 [-0.80,0.63]	-0.13 [-0.84,0.57]
Self-regulation skills (B [95% CI])	Active2Gether-Light	Reference	Reference	Reference	Reference
	Active2Gether-Full	0.45 [-1.53,2.43]	0.40 [-1.65,2.45]	0.26 [-1.80,2.32]	0.39 [-1.69,2.48]
Satisfaction (OR [95% CI])	Active2Gether-Light	Reference	Reference	Reference	Reference
	Active2Gether-Full	1.31 [0.33,5.23]	1.58 [0.38,6.69]	1.15 [0.29,4.57]	1.69 [0.39,7.34]

Note. Linear regression analyses are presented with regression coefficient (B) [95% confidence interval (95%CI)] and logistic regression analyses with odds ratio (OR) [95% confidence interval (95%CI)], and all analyses were adjusted for levels of physical activity at baseline and time between baseline and post-intervention follow-up.

Model 0: $y = B_0 + B_1$*Determinant at post-intervention + B_2* Determinant at baseline + B_3* Time until post-intervention follow-up (days)

Model 1: Model 0 + B_4* BMI (kg/m^2)

Model 2: Model 0 + B_4*Student (yes/no);

Model 3: Model 0 + B_4* Student(yes/no) + B_5* Meeting physical activity (PA) guidelines at baseline (yes/no)
